# Cirugía de revascularización de miocardio usando arteria mamaria interna bilateral. Resultados a mediano plazo.

**DOI:** 10.47487/apcyccv.v1i1.11

**Published:** 2020-03-30

**Authors:** Julio Castillo, Josías Ríos

**Affiliations:** 1 Médico residente de Cirugía de Tórax y Cardiovascular - Instituto Nacional Cardiovascular - INCOR EsSalud. Lima, Perú. Instituto Nacional Cardiovascular - INCOR EsSalud Lima Perú; 2 Servicio de Cirugía Cardiovascular Adultos - Instituto Nacional Cardiovascular - INCOR EsSalud. Lima, Perú. Servicio de Cirugía Cardiovascular Adultos Instituto Nacional Cardiovascular - INCOR EsSalud Lima Perú

**Keywords:** cirugía de revascularización de miocardio, doble arteria mamaria interna, coronary artery bypass graft, bilateral internal mammary artery

## Abstract

**Introducción:**

: La cirugía de revascularización quirúrgica de miocardio es una de las principales estrategias en el tratamiento de la enfermedad coronaria. Está ampliamente demostrado el beneficio del uso de la arteria mamaria interna (AMI) izquierda para revascularizar la arteria descendente anterior, mejorando la supervivencia a largo plazo. Por otro lado, existen aún corrientes variables acerca del uso de dos arterias mamarias internas, a pesar de que también está demostrada la superioridad del uso de dos arterias mamarias sobre una.

**Metodología:**

: Investigación retrospectiva de las cirugías de revascularización quirúrgica de miocardio con AMI bilateral, realizadas en el Instituto Nacional Cardiovascular - INCOR EsSalud entre enero de 2012 y junio de 2018. Los objetivos fueron determinar la tasa de mortalidad y definir la tasa de eventos adversos mayores cardiovasculares en un seguimiento a mediano plazo de 30 meses.

**Resultados:**

121 pacientes fueron operados con AMI bilateral. A todos los pacientes se les realizó disección de arteria mamaria con técnica esqueletizada. Hubo una muerte intrahospitalaria a causa de mediastinitis. Los eventos cardiovasculares mayores se presentaron en 5.8 % de los pacientes (muerte 0.8%, stroke 0%, infarto de miocardio perioperatorio 1.6%, necesidad de nueva intervención coronaria 3.3%). La incidencia de mediastinitis y/o reconstrucción esternal fue de 0.8%.

**Conclusiones:**

La revascularización quirúrgica de miocardio con AMI bilateral es un procedimiento seguro, con bajas tasas de mortalidad y de eventos cardiovasculares mayores en un seguimiento a 30 meses.

## Abstract (en)

**Introduction:**

Coronary artery bypass graft (CABG) surgery is one of the main strategies in the treatment of coronary heart disease. The benefit of using the left internal mammary artery (IMA) to revascularize the anterior descending artery is greatly demonstrated, improving long-term survival in patients receiving this graft. On the other hand there are variable currents about the use of two internal mammary arteries, although several studies have already demonstrated the superiority of the use of two mammary arteries over one.

**Methodology:**

Retrospective study of Coronary artery bypass graft surgeries with bilateral mammary artery, performed at Instituto Nacional Cardiovascular - INCOR EsSalud between January 2012 and June 2018. The objective was to determine the mortality rate and major adverse cardiovascular events in a medium term follow-up of 30 months.

**Results:**

121 patients were operated with bilateral internal mammary artery. All patients underwent dissection of mammary artery with skeletonized technique. There was one in-hospital death due to mediastinitis. Major adverse cardiovascular events occurred in 5.8% of patients (death 0.8%, stroke 0%, perioperative myocardial infarction 1.6%, need for new coronary intervention 3.3%). The incidence of mediastinitis and/or sternal reconstruction was 0.8%.

**Conclusions:**

: CABG with bilateral IMA is a safe procedure, with low mortality rates and low major adverse cardiovascular events in a 30-month follow-up.

Pese a los grandes avances en la colocación de stents coronarios, la cirugía de revascularización de miocardio (RVM) permanece como la principal forma de revascularización en casos de enfermedad coronaria multiarterial y enfermedad de tronco de coronaria izquierda; el bypass de la arteria mamaria interna izquierda (AMII) a la arteria descendente anterior (DA) es el más importante a realizar en estos casos.^1^^-^^3^ Muchos pacientes tienen lesiones adicionales a las de la DA, en vasos que también necesitan ser revascularizados, para realizar todos estos bypass se usa también la arteria radial y la vena safena interna (VSI). ^(^^4^^-^^6^

Los resultados favorables del bypass AMII a DA han sido ampliamente demostrados. Debido a esto, muchos grupos quirúrgicos utilizan la arteria mamaria derecha (AMD) como injerto coronario adicional, y diversos estudios ya han demostrado mejor evolución en pacientes que reciben dos arterias mamarias. Se ha demostrado angiográficamente y con angiotomografia coronaria, tasas de permeabilidad del 98% a los 7 días y 95% a los 10 años. ^(^^7^^-^^9^

Las tasas de mortalidad a un año son similares para el uso de una o ambas arterias mamarias (AMI unilateral: 2.3%, AMI bilateral: 2.5%), sin embargo el uso de AMI bilateral se asocia a un incremento del 1.3% en las tasas de infección y reconstrucción esternal por dehiscencia o por mediastinitis. Los eventos adversos mayores cardiovasculares como infarto de miocardio, ictus y mediastinitis suelen tener tasas de 2,2%, 0,9% y 2.2%, respectivamente cuando se usan dos arterias mamarias; además, se describe una tasa de necesidad de reintervención coronaria de urgencia por cirugía de 0.7% y percutánea en 2.1%.^10^

Dos factores que suelen limitar el uso de dos arterias mamarias son, en primer lugar, el incremento del tiempo operatorio por la necesidad de disección de la arteria mamaria derecha y, en segundo, su asociación con mediastinitis sobre todo en pacientes diabéticos. Dorman et al. ^(^^11^ realizaron un estudio comparando el uso de AMI unilateral versus AMI bilateral en pacientes diabéticos (646 no diabéticos versus 461 diabéticos). La mortalidad a 30 días fue de 2.4% vs 3.15% (p=0.279) y la tasa de infección esternal fue de 1.7% vs 3.1% (p = 0.179), respectivamente. Sin embargo, en el seguimiento se evidenció que la sobrevida media de los pacientes con AMI unilateral fue de 9.8 años vs AMI bilateral 13.1 años (p = 0.001). El uso de ambas arterias mamarias se asoció a mayor sobrevida en el análisis de regresión de Cox (p = 0.003). ^(^^11^

Actualmente se recomienda el uso de AMI bilateral en pacientes menores de 70 años, sobrevida mayor a 10 años, inclusive en diabéticos que no tengan obesidad mórbida y con diabetes mellitus controlada definida como hemoglobina glicosilada (HbA1c) < 7%.^12^^,^^13^ La RVM con dos arterias mamarias es una cirugía relativamente nueva en nuestro centro.

## Material y Método

Realizamos un estudio descriptivo, retrospectivo de las cirugías de RVM realizadas en el Instituto Nacional Cardiovascular - INCOR EsSalud entre enero de 2012 y junio de 2018. Incluimos a todos los pacientes en los que se utilizó AMI bilateral; se excluyeron los pacientes que fueron sometidos a cirugía de urgencia, choque cardiogénico, reoperación y en aquellos en los que los datos de la historia clínica no eran claros o completos.

Los objetivos fueron determinar la mortalidad y definir la tasa de eventos adversos mayores cardiovasculares a 30 meses de seguimiento. Definimos los eventos cardiovasculares mayores con 4 indicadores, muerte, infarto agudo de miocardio (IAM) perioperatorio, stroke y la necesidad de reintervención coronaria (percutánea o quirúrgica). 

Todos los pacientes fueron tratados en el postoperatorio inmediato en la unidad de cuidados intensivos postquirúrgicos, donde recibieron evaluación clínica diaria, controles ecocardiográficos en las primeras 48 horas del postoperatorio, medición de enzimas cardíacas y toma de electrocardiograma en el postoperatorio inmediato (en la primera hora del postoperatorio) y luego diariamente durante la hospitalización. 

Para el análisis estadístico se utilizaron medidas de tendencia central y de dispersión según el tipo de variable estudiada. Para esto se usó el software STATA 16.

## Resultados

### Características de la población

**Figura 1:**
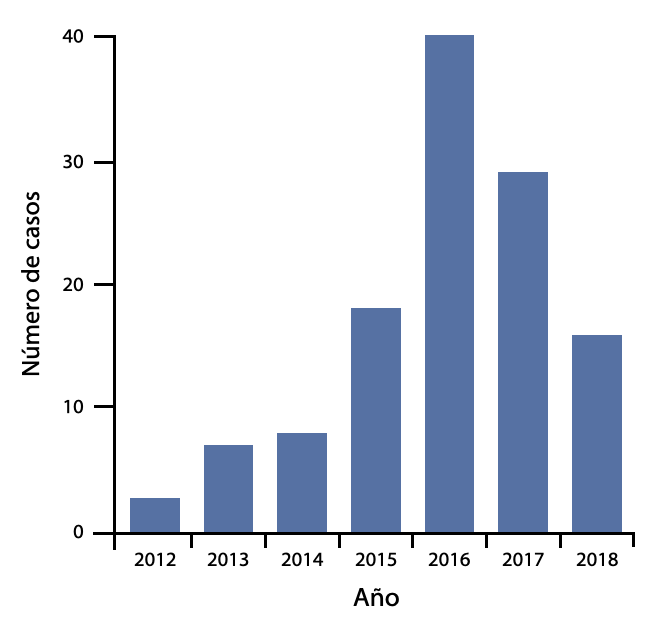
Número de cirugías por año

Desde enero de 2012 a junio de 2018, a 135 pacientes se les realizo RVM con AMI bilateral en el Instituto Nacional Cardiovascular - INCOR EsSalud, de los cuales solo 121 cumplieron con los criterios de selección. El número de pacientes por año se presentan en la Figura 1. Las características basales de la población se muestran en la Tabla 1.

**Tabla 1:** Características de la población

Edad	58.3	(± 9.7)
Mayores de 65 años	31	(25.6)
Sexo masculino	113	(93.4)
Diabetes mellitus 2	32	(26.4)
Hipertensión arterial	66	(54.5)
Dislipidemia	56	(46.3)
Infarto de miocardio previo	41	(33.9)
Tabaquismo	40	(33.0)
Angina inestable	6	(4.9)
Euroscore II	1.46	(± 1.4)
FEVI preoperatoria	58.5	(± 14.3)
FEVI < 40%	7	(5.8)
FEVI postoperatoria	57.8	(12.9)

Se reporta medias (desviación estándar) y frecuencias (porcentaje) para variables cuantitativas y categóricas, respectivamente.

FEVI: Fracción de eyección del ventrículo izquierdo.

### Características de la cirugía

De las 121 cirugías, 110 (90.9%) se realizaron con circulación extracorpórea (CEC) y 11 cirugías (9.1%) se realizaron sin CEC. El tiempo operatorio promedio total fue de 288.14 minutos. En las RVM con CEC el tiempo de CEC promedio fue de 91.7 minutos y el tiempo promedio de pinzamiento aórtico fue de 78 minutos. (Tabla 2)

**Tabla 2:** Características de las cirugías

Tipo de RVM		
RVM con CEC	110	(90.9)
Tiempo de CEC	91.6	(± 39.5)
Tiempo de pinzamiento	78.0	(± 28.2)
RVM sin CEC	11	(9.1)
Tipos de anastomosis		
AMII hacia DA, AMID hacia Mg	45	(37.2)
AMIII hacia DA, AMID hacia Dg	27	(22.3)
AMID hacia DA, AMID hacia CD	11	(9.0)
AMID hacia DA, AMII hacia Mg	38	(31.4)
Uso de VSI	62	(51.2)
Uso de arteria radial	17	(14.0)
RVM arterial completa	59	(48.8)

Se reporta medias (desviación estándar) y frecuencias (porcentaje) para variables cuantitativas y categóricas, respectivamente.

RVM: revascularización miocárdica; CEC: circulación extracorpórea; AMII: arteria mamaria interna izquierda; AMID: arteria mamaria interna derecha; DA: descendente anterior; Mg: marginal; Dg: diagonal; CD: coronaria derecha; VSI: vena safena interna.

### Técnica de disección y anastomosis

**Figura 2:**
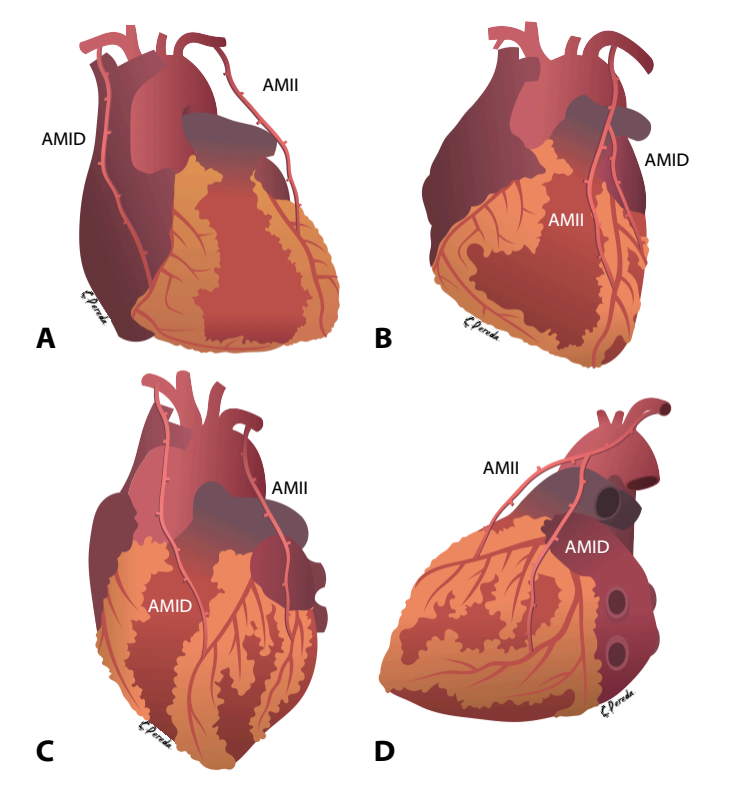
Técnica de Anastomosis

Se disecaron ambas arterias mamarias con técnica esqueletizada. La mayoría de las RVM se realizaron utilizando como injerto libre la AMID, para luego anastomosarla a la AMII, creando así un injerto compuesto en «Y invertida». De esta forma realizamos los puentes AMII hacia DA y AMID hacia Mg en 45 pacientes (37.2%) y AMII hacia DA, AMID hacia Dg en 27 pacientes (22.3%). En algunos casos se realizaron bypass sin desinsertar la AMID de su origen en la arteria subclavia; así se realizaron los bypass AMII hacia DA, AMID hacia coronaria derecha en 11 pacientes (9%) y AMID hacia DA, AMII hacia Mg en 38 (31.4%). Para revascularizar los vasos faltantes se usaron la arteria radial en 17 pacientes (14%) y la vena safena interna en 62 pacientes (51.27%). Además, en 59 pacientes (48.8%) se realizó revascularización completa con injertos arteriales (Figura 2).

A: AMII hacia DA, AMID hacia CD B: AMII hacia DA, AMID hacia Dg (injerto en «Y invertida») C: AMID hacia DA; AMII hacia Mg D: AMII hacia DA, AMID hacia Mg (injerto en «Y invertida») AMII: arteria mamaria interna izquierda; AMID: arteria mamaria interna derecha; DA: descendente anterior; Mg: marginal; Dg: diagonal; CD: coronaria derecha

### Mortalidad y complicaciones

La mortalidad a los 30 meses de seguimiento fue de 0.8%, que corresponde a una muerte debida a mediastinitis por E. Coli a los 30 días de la cirugía, teniendo en cuenta que esta paciente tuvo como condición previa una infección del tracto urinario. Los eventos cardiovasculares mayores que se presentaron a los 30 meses de seguimiento ocurrieron en 7 pacientes (5.8 %). No hubo casos de stroke. Sin embargo, 2 pacientes (1.6%) presentaron criterios de infarto agudo de miocardio perioperatorio. En estos casos, al realizar la coronariografía se encontraron los bypass con las arterias mamarias permeables, por lo que no se requirió angioplastia o cirugía de rescate. Se asumió que la causa del infarto fue por vasos no revascularizados o por una deficiente protección miocárdica durante la cirugía. En 4 pacientes hubo la necesidad de reintervención coronaria debido a que se vio afectado el puente de la AMII hacia la DA; en 3 de estos (2.5%) casos mediante intervencionismo y en uno (0.8%) mediante una nueva cirugía coronaria en el que se utilizó un injerto de arteria radial para hacer un puente hacia la arteria descendente anterior. Se encontró además un caso de mediastinitis, y dehiscencia esternal que requirió reconstrucción del mismo hueso. (Tabla 3)

**Tabla 3:** Evolución postoperatoria

Evolución en UCI		
Estancia (días)	2.5	(± 2.0)
Evento adverso cardíaco mayor	7	(5.8)
Muerte	1	(0.8)
Infarto perioperatorio	2	(1.6)
Stroke	0	(0)
Reintervención coronaria	4	(3.3)
Angioplastía percutánea	3	(2.5)
Cirugía coronaria	1	(0.8)
Otras complicaciones		
Mediastinitis	1	(0.8)
Insuficiencia renal	3	(2.5)
Sangrado excesivo	7	(5.8)
Infección superficial de la herida	9	(7.4)

Se reporta medias (desviación estándar) y frecuencias (porcentaje) para variables cuantitativas y categóricas, respectivamente.

UCI: unidad de cuidados intensivos

Una de las complicaciones que se presentó con relativa frecuencia fue el sangrado postoperatorio excesivo. 7 pacientes (5.8%) requirieron ingresar a sala de operaciones para revisión de hemostasia.

## Discusión

Las guías clínicas de revascularización actualmente recomiendan el uso de AMII bilateral en los pacientes menores de 70 años, o en aquellos con una expectativa de vida mayor a 10 años. ^(^^12^^)^ En nuestro estudio 31 pacientes tuvieron más de 65 años, pero todos con una esperanza de vida estimable por encima de 10 años. 

La diabetes no controlada y la obesidad se asocian per se a mayor riesgo de desarrollar mediastinitis postcirugía cardíaca. ^(^^13^^-^^14^ Aunque, si bien es cierto, la cirugía de RVM con AMI bilateral se asocia a un ligero incremento de mediastinitis, se justifica y con creces este riesgo, porque las tasas de sobrevida son mayores cuando la RVM se realiza con AMI bilateral, y la diabetes mellitus aparentemente no incrementa este riesgo. ^(^^15^^-^^17^ Sin embargo, cabe mencionar que los trabajos de investigación incluyen a pacientes diabéticos controlados con niveles de HbA1c < 7%.^11^ En nuestra investigación incluimos 32 pacientes con antecedente de diabetes mellitus (26.4% de la población), pero todos eran diabéticos controlados con niveles de HbA1c < 7%. Además, 74 pacientes (61.1%) pacientes tenían sobrepeso/obesidad, mas no obesidad mórbida (IMC > 40). 

Como se mencionó antes, se presentó un solo caso de mediastinitis y dehiscencia esternal. Sin embargo, los pacientes diabéticos deben tener niveles de glucemia y de HbA1c normales antes de la cirugía, por lo que podría ser adecuado que aquellos pacientes no controlados sean hospitalizados varios días antes de la fecha de la cirugía para mejorar sus niveles de glucemia y de HbA1c. ^(^^12^

Se han descrito 2 técnicas de disección para las AMI: la «esqueletizada» y la «pediculada». En la primera, los vasos venosos mamarios se preservan íntegros e in situ; por el contrario, en la técnica pediculada, la arteria mamaria se diseca junto a los vasos venosos y con el tejido circundante. Con relación a esto, Benedetto et al, en un estudio de 219 pacientes con mediastinitis postquirúrgica, probaron que cuando se usa AMI unilateral no existe diferencia en relación con el porcentaje de mediastinitis con cualquiera de las técnicas; sin embargo, cuando se usa AMI bilateral la técnica de disección pediculada incrementa el riesgo de presentar mediastinitis posquirúrgica (OR = 1.80; IC 95%: 1.23-2.63). ^(^^14^^)^ De igual forma, Barros Oliveira et al. realizaron un metaanálisis de 2,633 pacientes, demostraron que cuando se disecaron ambas AMI la técnica esqueletizada tenía menor incidencia de mediastinitis (OR = 0.327; IC 95%: 0.217-0.492; p < 0.001). ^(^^18^ Otros investigadores^19^ han encontrado resultados parecidos. Debido a esto, en todos nuestros pacientes se realizó la técnica de disección esqueletizada y solo tuvimos un caso de mediastinitis que fue el paciente que falleció. 

Nuestra mortalidad fue de 0.8% (1 paciente dentro de los 30 meses de seguimiento). La mortalidad descrita en por Taggart^17^ en el estudio ART es de 2.5% a los 10 años, siendo muy similar la mortalidad para los pacientes con una sola mamaria que para los que recibieron doble mamaria; y la mortalidad descrita por Adelborg et al. en un seguimiento de 30 años fue de 3.2%, situándose nuestra mortalidad por debajo de estas cifras, a un seguimiento de 30 meses. ^(^^20^

Los eventos cardiovasculares mayores incluida la muerte, stroke, IAM posquirúrgico y nueva intervención coronaria, se presentaron en el 5.8% de los pacientes, 2 IAM posquirúrgicos, 4 reintervenciones coronarias y una muerte. En relación con la mortalidad, otros autores reportan tasas entre 1-2% con el uso de AMI bilateral, no habiendo mayores diferencias con la AMI unilateral. ^(^^21^^,^^22^

La incidencia de stroke se ha descrito entre 0.9 y 3% en algunos estudios, pero en el nuestro no tuvimos dicha complicación. ^(^^10^ En relación con el infarto de miocardio perioperatorio, algunos estudios han descrito una incidencia de hasta el 10% en la cirugía de RVM en general. Sin embargo, en estudios de RVM con AMI bilateral ha sido descrito en menos del 4%^22^^,^^23^. Otros estudios en un seguimiento a 10 años incluso han descrito tasas de IMA perioperatorio de 2.2%.^10^ En nuestra serie, la incidencia fue del 1.65%. Lo más preocupante es nuestra alta tasa de reoperación por sangrado excesivo (5.8%), ya que otros autores reportan tasas mucho más bajas como 1.15%, esto posiblemente debido a la curva de aprendizaje y al hecho de tener dos lechos mamarios potencialmente sangrantes. ^(^^22^^,^^24^^)^ En cuanto a la necesidad de reintervención coronaria la bibliografía describe tasas de 0.7% para reintervenciones quirúrgicas y 2.1% para reintervenciones percutáneas. Nuestras tasas de reintervenciones fueron de 2.5% por vía percutánea y 0.8% por cirugía, estando dentro de rangos muy similares a los descritos. ^(^^25^

## Conclusión

La RVM con AMI bilateral es un procedimiento seguro, inclusive en pacientes diabéticos controlados, con bajas tasas de mortalidad y de eventos cardiovasculares mayores a mediano plazo.

## Financiación:

Ninguna
